# Supplementary material for: Divergent Effects of Peptidoglycan Carboxypeptidase DacA on Intrinsic β-Lactam and Vancomycin Resistance
Source: Microbiol Spectr. 2022 Jun 27;10(4):e01734-22. doi: 10.1128/spectrum.01734-22 (PMC9430164; doi:10.1128/spectrum.01734-22)
Supplement: Supplemental file 1 — Fig. S1-S6; Tables S1 and S2. Download spectrum.01734-22-s0001.pdf, PDF file, 0.5 MB [file spectrum.01734-22-s0001.pdf]

Supplementary materials

1. Supplementary Figures

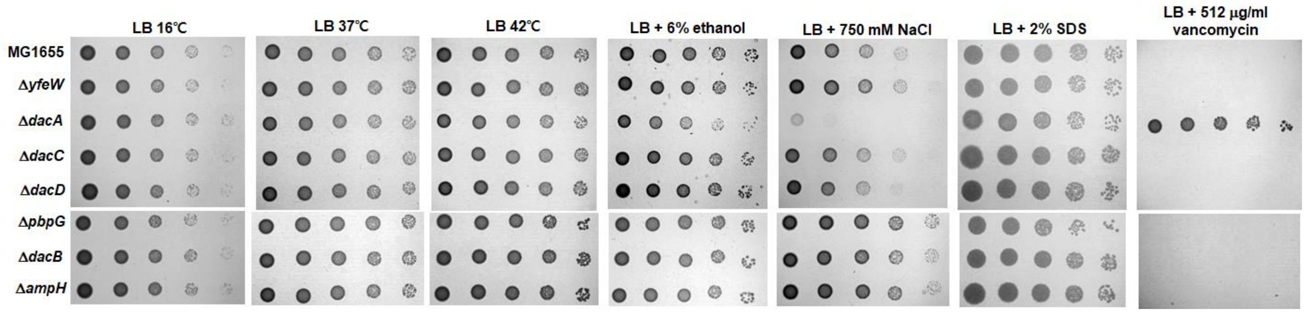

FIG S1 Envelope stress-related phenotypes of PG carboxypeptidase-defective mutants. The wild-type and indicated mutant cells were serially diluted from 10^8 to 10^4 cells/ml in 10-fold steps and spotted onto LB plates or LB plates containing indicated compounds.

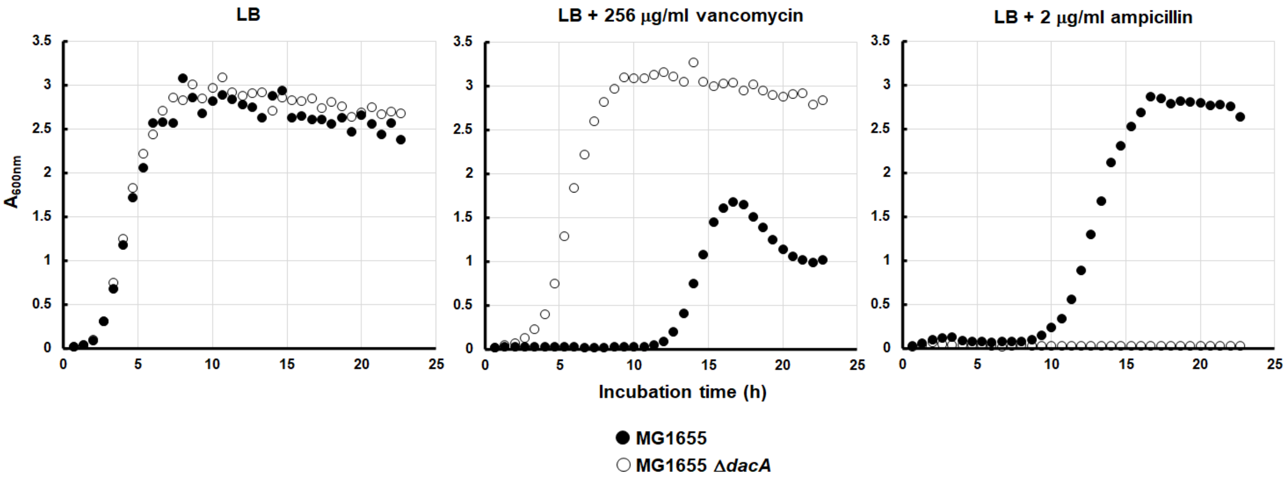

FIG S2 The effect of vancomycin and ampicillin on the growth of the *dacA* mutant. The MG1655 or *dacA* mutant cells grown in LB overnight were inoculated in LB medium or LB medium with indicated concentrations of antibiotics. Cell growth was recorded by measuring the optical density at 600 nm: closed circles, MG1655; open circles, MG1655 Δ*dacA*.

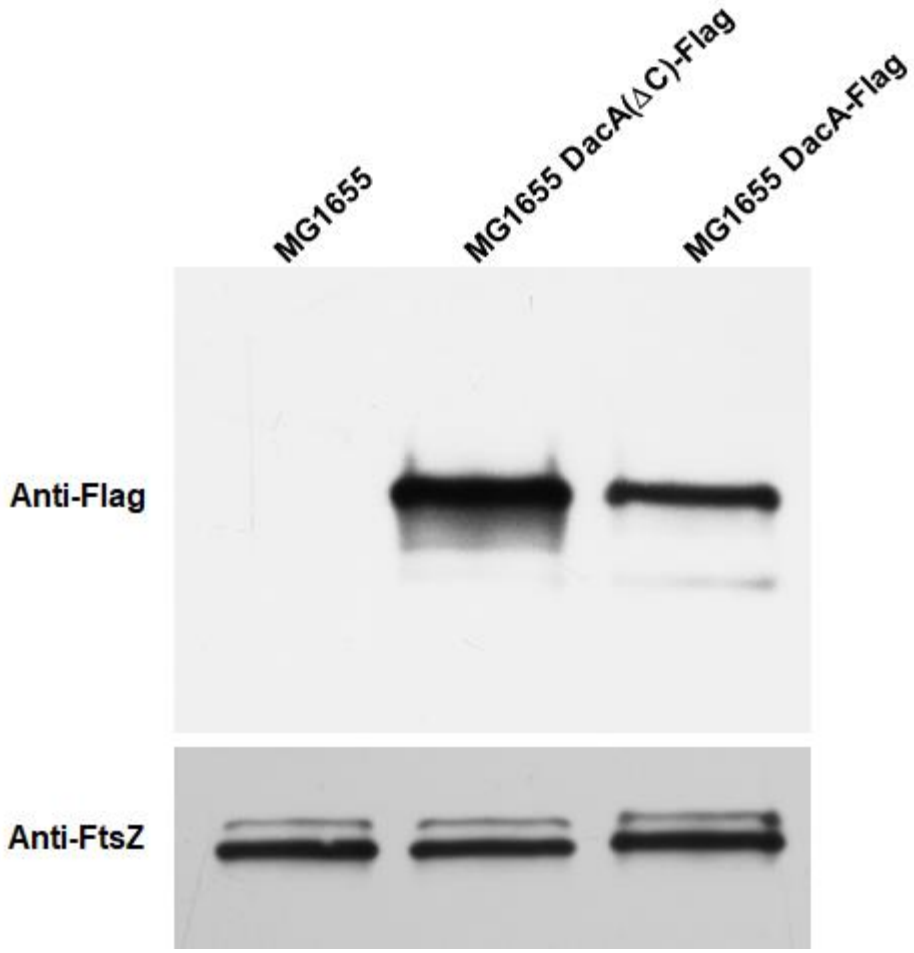

FIG S3 Intracellular protein levels of DacA and DacA(ΔC). Western blot analysis with anti-Flag and anti-FtsZ antibodies was performed using 5 x 10^7 cells of indicated strains grown in the LB to the early exponential phase (OD_{600nm} = 0.4). FtsZ was used as the loading control.

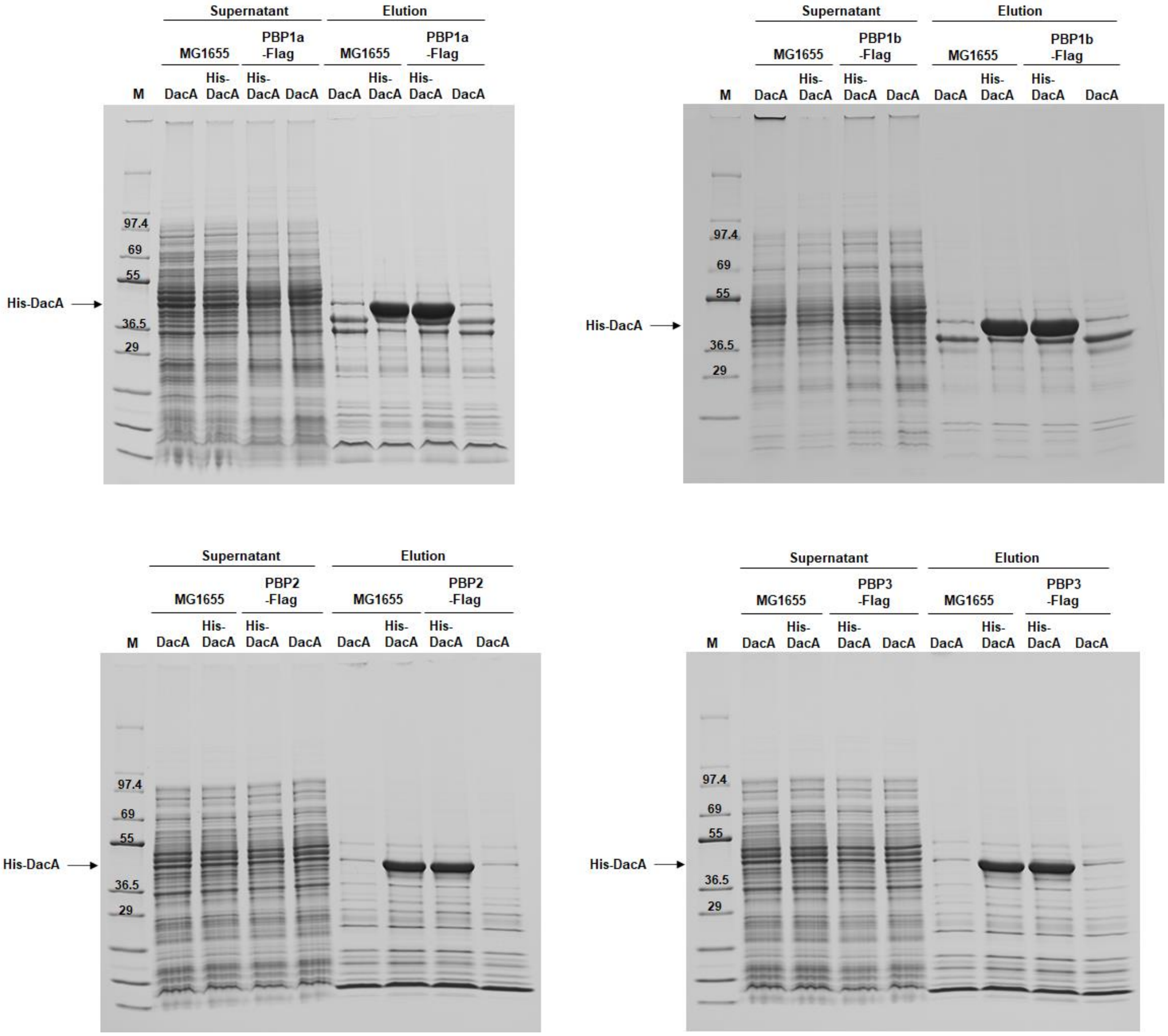

Fig S4 SDS-PAGE gels of pull-down experiments using His-DacA. The supernatant of MG1655 cells or MG1655 cells with three Flag-tags fused to the C-terminus of the indicated chromosomal PBP was mixed with the supernatant of ER2566 cells harboring pET-based plasmid expressing His-tagged DacA or non-tagged DacA. After pull-down experiments, input (Supernatant) and output (Elution) samples were separated in 4–20% gradient Tris-glycine polyacrylamide gels (KOMA biotech, Korea) and stained with Coomassie brilliant blue. Lane M indicates EzWayTM Protein Blue MW Marker (KOMA Biotech., Korea).

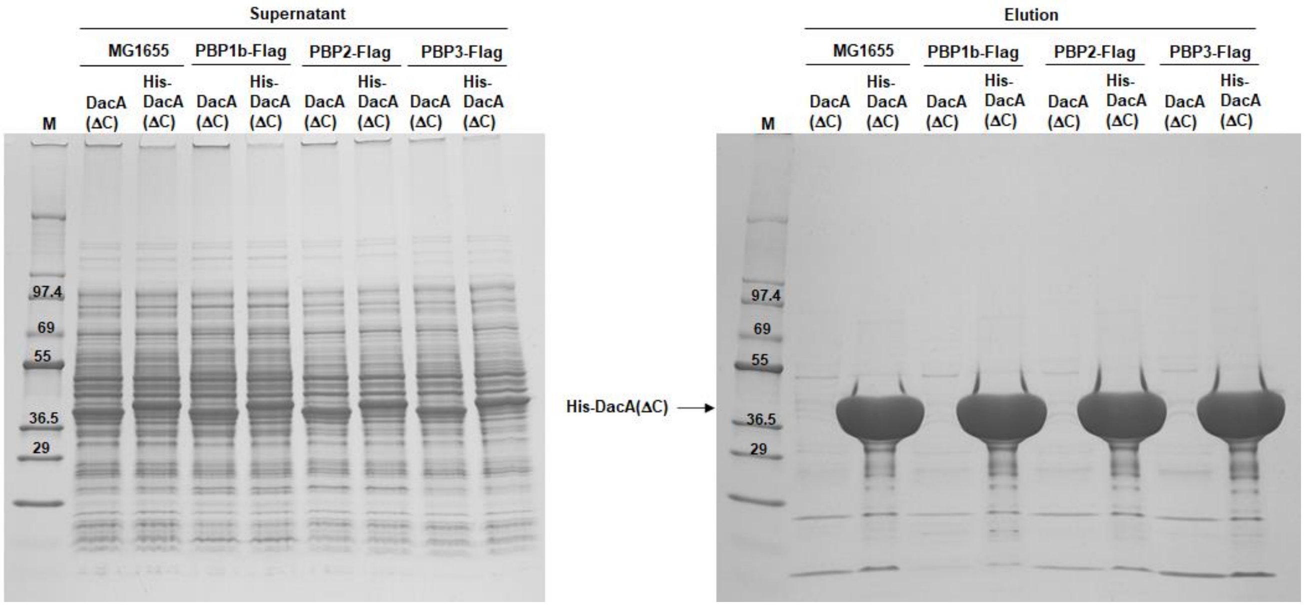

Fig S5 SDS-PAGE gels of pull-down experiments using His-DacA(ΔC). The supernatant of MG1655 cells or MG1655 cells with three Flag-tags fused to the C-terminus of the indicated chromosomal PBP was mixed with the supernatant of ER2566 cells harboring pET-based plasmid expressing His-tagged DacA(ΔC) or non-tagged DacA(ΔC). After pull-down experiments, input (Supernatant) and output (Elution) samples were separated in 4–20% gradient Tris-glycine polyacrylamide gels (KOMA biotech, Korea) and stained with Coomassie brilliant blue. Lane M indicates EzWayTM Protein Blue MW Marker (KOMA Biotech., Korea).

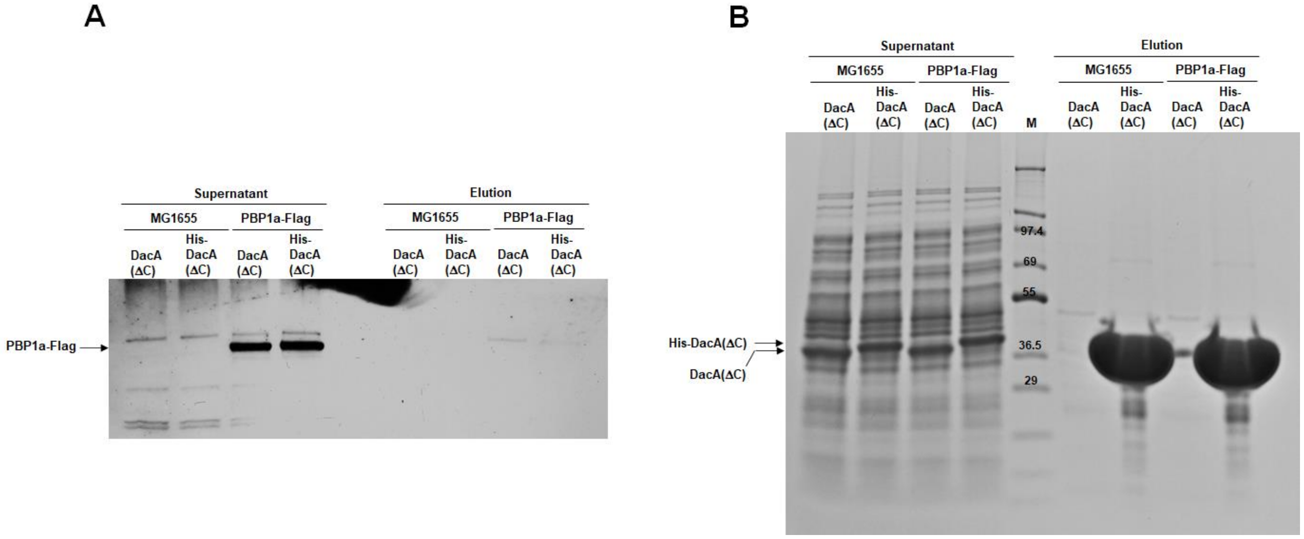

Fig S6 The importance of the C-terminal domain of DacA for its interaction with PBP1a. (A) The supernatant of MG1655 cells or MG1655 cells with three Flag-tags fused to the C-terminus of the indicated chromosomal PBP1a was mixed with the supernatant of ER2566 cells harboring pET-based plasmid expressing His-tagged DacA(ΔC) or non-tagged DacA(ΔC). After pull-down experiments, the amount of input (Supernatant) and output (Elution) PBP1a was measured by western blot using monoclonal antibody against Flag-tag. (B) After pull-down experiments, input (Supernatant) and output (Elution) samples were separated in 4–20% gradient Tris-glycine polyacrylamide gels (KOMA biotech, Korea) and stained with Coomassie brilliant blue. Lane M indicates EzWayTM Protein Blue MW Marker (KOMA Biotech., Korea).

2. Supplementary Tables

Supplementary Table S1. *Escherichia coli* and *Bacillus subtilis* strains and plasmids used in this study.

Strain or plasmid	Genotype or phenotype	Source or Reference
**Strains**		
MG1655	F^- λ^- *ilvG*$^-$ *rfb*-50 *rph*-1. Wild type *E. coli* K-12	(Blattner *et al.*, 1997)
Bacillus subtilis		(Ji *et al.*, 2018)
MG1655 Δ*dacA*	MG1655 *dacA*:: *frt*	This study
MG1655 Δ*dacB*	MG1655 *dacB*:: *frt*	(Park *et al.*, 2020)
MG1655 Δ*dacC*	MG1655 *dacC*:: *frt*	This study
MG1655 Δ*dacD*	MG1655 *dacD*:: *frt*	This study
MG1655 Δ*pbpG*	MG1655 *pbpG*:: *frt*	(Park *et al.*, 2020)
MG1655 Δ*ampH*	MG1655 *ampH*:: *frt*	(Park *et al.*, 2020)
MG1655 Δ*yfeW*	MG1655 *yfeW*:: *frt*	This study
MG1655 Δ*dacA* Δ*dacD*	MG1655 *dacA*::*frt dacD*::Km^r	This study
MG1655 Δ*dacA* Δ*dacD* Δ*ampH*	MG1655 *dacA*::*frt dacD*:: *frt ampH*::Km^r	This study
MG1655 Δ*dacA* Δ*dacD* Δ*ampH* Δ*dacB*	MG1655 *dacA*::*frt dacD*:: *frt ampH*:: *frt dacB*::Km^r	This study
MG1655 Δ*ldtABC*	MG1655 *ldtA*::*frt ldtB*:: *frt ldtC*:: *frt*	This study
MG1655 Δ*ldtDE*	MG1655 *ldtD*::*frt ldtE*:: *frt*	This study
MG1655 Δ*lpp*	MG1655 *lpp*::*frt*	This study
MG1655 Δ*ldtDE* Δ*lpp*	MG1655 *ldtD*::*frt ldtE*:: *frt lpp*::*frt*	This study

MG1655 Δ*dacA* Δ*lpp*	MG1655 *dacA*:: *frt lpp*::*frt*	This study
MG1655 Δ*ompA* Δ*ompC* Δ*ompF*	MG1655 *ompF::frt ompC::frt ompA:: frt*	(Choi and Lee, 2019)
MG1655 Δ*ompA* Δ*ompC* Δ*ompF* Δ*dacA*	MG1655 *ompF::frt ompC::frt ompA:: frt dacA*:: *frt*	This study
MG1655 DacA(ΔC)-Flag	MG1655 DacA(1-385)-3xFLAG	This study
MG1655 PBP1a-Flag	MG1655 *mrcA*-3xFLAG, Cm^R	This study
MG1655 PBP1b-Flag	MG1655 *mrcB*-3xFLAG, Cm^R	This study
MG1655 PBP2-Flag	MG1655 *mrdA*-3xFLAG, Cm^R	This study
MG1655 PBP3-Flag	MG1655 *ftsI*-3xFLAG, Cm^R	This study
MG1655 DacA-Flag	MG1655 *dacA*-3xFLAG, Cm^R	This study
B. subtilis Δ*dacA*	*B. subtilis* Δ*dacA*	This study
B. subtilis Δ*dacB*	*B. subtilis* Δ*dacB*	This study
B. subtilis Δ*dacF*	*B. subtilis* Δ*dacF*	This study
ER2566	F-λ-*fhuA2* [*lon*] *ompT lacZ*::T7 gene 1 gal *sulA11* Δ(*mcrC-mrr*)*114*::*IS10* R(*mcr-73*::*miniTn10*-TetS)2 R(*zgb-210*::*Tn10*)(TetS) *endA1* [*dcm*]	New England Biolabs
**Plasmids**		
pBAD24	Expression vector under control of arabinose-inducible promoter, Amp^r	Addgene
pBAD24(cm)	pBAD24-based expression vector, Cm^r	(Park *et al.*, 2020)
pBAD-Flag	pBAD24(cm)-based expression vector for the 3xFlag tag, Cm^r	(Park *et al.*, 2020)

pBAD-DacA	pBAD24(cm)-based expression vector for DacA, Cm^r	This study
pBAD-DacB	pBAD24(cm)-based expression vector for DacB, Cm^r	(Park *et al.*, 2020)
pBAD-DacC	pBAD24(cm)-based expression vector for DacC, Cm^r	This study
pBAD-DacD	pBAD24(cm)-based expression vector for DacD, Cm^r	This study
pBAD-YfeW	pBAD24(cm)-based expression vector for YfeW, Cm^r	This study
pBAD-PbpG	pBAD24(cm)-based expression vector for PbpG, Cm^r	(Park *et al.*, 2020)
pBAD-AmpH	pBAD24(cm)-based expression vector for AmpH, Cm^r	(Park *et al.*, 2020)
pBAD-PBP1a-Flag	pBAD24(cm)-based expression vector for PBP1a fused with the 3xFlag tag at the C-terminus, Cm^r	This study
pBAD-PBP1b-Flag	pBAD24(cm)-based expression vector for PBP1b fused with the 3xFlag tag at the C-terminus, Cm^r	This study
pBAD-PBP2-Flag	pBAD24(cm)-based expression vector for PBP2 fused with the 3xFlag tag at the C-terminus, Cm^r	This study
pBAD-PBP3-Flag	pBAD24(cm)-based expression vector for PBP3 fused with the 3xFlag tag at the C-terminus, Cm^r	This study
pBAD-Flag-FRT-Kan	pBAD24(cm)-based expression vector containing the 3xFlag tag, FRT-Km^r, Cm^r	This study
pBAD-DacA(ΔC)-Flag-FRT-Kan	pBAD24(cm)-based expression vector for DacA(1-385) fused with the 3xFlag tag C-terminus, FRT-Km^r, Cm^r	This study
pKD13	Template plasmid for the amplification of the kanamycin-resistance gene bordered by FRT sites, Km^r	(Datsenko and Wanner, 2000)
pKD46	λ Red recombinase expression plasmid Ts replicon, Amp^r	(Datsenko and Wanner, 2000)
pCP20	FLP helper plasmid Ts replicon, Amp^r, Cm^r	(Datsenko and Wanner, 2000)

pET28a	Expression vector under control of T7 promoter, Km^r	Novagen
pET24a	Expression vector under control of T7 promoter, Km^r	Novagen
pET24a-DacA	pET24a-based expression vector for DacA(30-403), Km^r	This study
pET24a-DacA(ΔC)	pET24a-based expression vector for DacA(30-385), Km^r	This study
pET24a-PBP1a	pET24a-based expression vector for PBP1a, Km^r	This study
pET24a-PBP1b	pET24a-based expression vector for PBP1b, Km^r	This study
pET28a-DacA	pET28a-based expression vector for DacA(30-403) with N-terminal 6 histidines, Km^r	This study
pET28a-DacA(ΔC)	pET28a-based expression vector for DacA(30-385) with N-terminal 6 histidines, Km^r	This study
pET28a-NDM-1	pET28a-based expression vector for NDM-1 with N-terminal 6 histidines, Km^r	This study
pRE1	Expression vector under control of promoter, Amp^r	(Reddy *et al.*, 1989)
pRE1(kan)	Expression vector under control of promoter, Km^r	This study
pRE1-DacA	pRE1-based expression vector for DacA, Km^r	This study
pRE1-DacA(ΔC)	pRE1-based expression vector for DacA(1-385), Km^r	This study
pRE1-DacA(S73G)	pRE1-based expression vector for DacA(S73G), Km^r	This study
pRE1-DacA(K242R)	pRE1-based expression vector for DacA(K242R), Km^r	This study

Supplementary Table S2. Oligonucleotides used in this study

Name	Oligonucleotide sequence (5’–3’)	Use(s)
DacA-FRT-F	CTATAGTAGGGCACTTTTTTAATTCCATCACG GATGTCGTAGTTCAGACCGTGTAGGCTGGAG CTGCTTC	
DacA-FRT-R	GGGTTAACGGTTTCAAAGAAACGGAAGCCCC AGGTTAGCAGTTTTTTACTATTCCGGGGATCC GTCGACC	
DacC-FRT-F	CATACGGCGATATAACGTATTTTTTTTGAATG GATACTCGGGTGGCATTTGTGTAGGCTGGAG CTGCTTC	
DacC-FRT-R	AAGGTGGCATCAGGTTTAATTGGCGTCACGG TTTCAAAGAAGCGGAAACCATTCCGGGGATC CGTCGACC	
DacD-FRT-F	AAGTATTTCCGTAAAAAGAACAGCTATTTGA AACTCCTGAGGGTTTGCTGGTGTAGGCTGGA GCTGCTTC	
DacD-FRT-R	TCCGTTCCGACCTTTTTCCCACGGTGCAAAAT TTGCACCGTAGTAAAGTTATTCCGGGGATCCG TCGACC	Deletion
YfeW-FRT-F	AACCAGATGGATCGCTGGATTAGCCAGCAAG TTGATGTCGGTTATCCCAGGTGTAGGCTGGAG CTGCTTC	
YfeW-FRT-R	CATTACCATTCACGCGCCAGCCGAGGCCAAA AGTGGCATCTTCCTTAGAGATTCCGGGGATCC GTCGACC	
LdtA-FRT-F	ATAAAAGCTATACTTAACGGATAGCTTTCGCG ACATAGGAAAGGGACATGGTGTAGGCTGGAG CTGCTTC	
LdtA-FRT-R	TTAAAACATCTGTCTTGAACCAGAACTAATTT GCACAGGCATTCCCGATCATTCCGGGGATCC GTCGACC	
LdtB-FRT-F	CTCTAATATTCTCAACCCAATGGCCTGCCAGG CACAAAATCTCGCTTAACGTGTAGGCTGGAG CTGCTTC	

LdtB-FRT-R	CAAACTGGGCTTCGGTGGTAGACAGCGGGTTATGGACTTCAATATAACGGATTCCGGGGATCCGTCGACC	
LdtC-FRT-F	TCAGGCTTATCTGTTTATTACAATAACCTTATATTTATTATGGATTTTTGGTGTAGGCTGGAGCTGCTTC	
LdtC-FRT-R	TGCATCACTTCAGCGTCAGTTTGTGCTGCATCTTTAAATGATTGCATTGCATTCCGGGGATCCGTCGACC	
LdtD-FRT-F	TAAAATAACAGCCTGGCTATTCAGAGTATGATAAAAACAGGGGGCAAGGGGTGTAGGCTGGAGCTGCTTC	
LdtD-FRT-R	CCCCAATCATGCTAATTATTACGACAACTGATTTCCCCGAACTACTTCATATTCCGGGGATCCGTCGACC	
LdtE-FRT-F	CGCCATCCGACGTTTCAGCGTGAGTCTCCGGCAAATACAGGAGGTTTACAGTGTAGGCTGGAGCTGCTTC	
LdtE-FRT-R	CCTGCCCGACGATACAACGCTTTATCGACTAACTTCTGATCTACAGCCTTATTCCGGGGATCCGTCGACC	
Lpp-FRT-F	AATACTTGTAACGCTACATGGAGATTAACTCAATCTAGAGGGTATTAATAGTGTAGGCTGGAGCTGCTTC	
Lpp-FRT-R	ACAAAAAAAATGGCGCACAATGTGCGCCATTTTTCACTTCACAGGTACTAATTCCGGGGATCCGTCGACC	
DacA-cfm-F	AAAGTCAGATGCCTGCCGGTAGTGCATTTG	Deletion confirm
DacA-cfm-R	TCAGAGGCGAACTCTTTACCTACTTTCAGT	
DacC-cfm-F	TGTGTGTGCGTTATTAATCACCAAACTTAT	
DacC-cfm-R	CGCTCTTATCACCAAACCAGACGCGCTGAG	
DacD-cfm-F	TTTTCTTGCACTTTATTCCAGCCAGTTCAT	
DacD-cfm-R	ATATTTTCTTTATCGCCATACCAGATGCGT	

YfeW-cfm-F	GAGAAAGCAGGGTTTAACGTCGAACGGCTT	
YfeW-cfm-R	CCAGCGTGCCAAACGTCGGCGTCATGGTGG	
LdtA-cfm-F	TGATTTGAACAGTTAAAAACGAAAAGTCTG	
LdtA-cfm-R	AGGCTTTTTGCTTTCTAATTACCAACGCTC	
LdtB-cfm-F	CTGGAATGAACTTATAATGCGCTTCCAATA	
LdtB-cfm-R	TCAGGGTAATTGGCACAATTTCCTGACCTT	
LdtC-cfm-F	GCACTATCCGACACTGTCACCATCCATAAT	
LdtC-cfm-R	GGCATCCCGGAACGGACATCCATCACATGT	
LdtD-cfm-F	CGTGAGTATTGGCGTTGTACAGGCAAGTCG	
LdtD-cfm-R	CCAGCAGTGACGGGGGCTGCAGAGAATCGC	
LdtE-cfm-F	AGGAGTGTTTTGGTTTCAGGTGAACATAAG	
LdtE-cfm-R	GTTGCTCCACTGCTCACCGAAACCGGATAC	
Lpp-cfm-F	GAGCGAACGATCAAAAATAAGTGCCTTCCC	
Lpp-cfm-R	CAAGGGAATATGTTACGCGTGACGCAGTAG	
PBP1a-3×Flag-F	ACGAGGTGGGAACGACCATTATCGATAATGGCGAGGCACAGGAATTGTTCGTCGACCTGCAGGATTATAA	Chromosomal 3×Flag tag
PBP1a-3×Flag-R	ACAAGTGCACTTTGTCAGCAAACTGAAAAGGCGCCGAAGCGCCTTTTTAATTACGCCCCGCCCTGCCACT	
PBP1b-3×Flag-F	AAGACAGCGACGGTGTAGCCGGTTGGATCAAGGATATGTTTGGTAGTAATGTCGACCTGCAGGATTATAA	
PBP1b-3×Flag-R	TGTTATTTTACCGGATGGCAACTCGCCATCCGGTATTTCACGCTTAGATGTTACGCCCCGCCCTGCCACT	

PBP2-3×Flag-F	ACACCGATCTGCCTGCGGAAAATCCAGCGGTTGCCGCAGCGGAGGACCATGTCGACCTGCAGGATTATAA	
PBP2-3×Flag-R	GAGATGGACTTTATCCCAGAATGTTTTTTTATTCGGATTATCCGTCATGATTACGCCCCGCCCTGCCACT	
PBP3-3×Flag-F	ATAAAAATGAATTTGTGATTAATCAAGGCGAGGGGACAGGTGGCAGATCGGTCGACCTGCAGGATTATAA	
PBP3-3×Flag-R	GTGCTCGCGAAGGTGCGTCTGGCACCCACGGAGCAAGAAGGTCGCGCAAATTACGCCCCGCCCTGCCACT	
DacA(ΔC)-FLAG-F	GCAATCGGGTAACGATGCTTGTGTCGCCAT	
DacA(ΔC)-FLAG-R	CTGATGCTTAGTATATGGGGACGGAAATTACACTTTCAAGTGTTTAATTTATTCCGGGGATCCGTCGACC	
PBP1a-cfm-F	GCCTATACCGCTACATCGAGCCACAACTGC	Chromosomal 3×Flag tag confirm
PBP1a-cfm-R	TAACGCGTTCACGCCGTATCCGGCATAAAC	
PBP1b-cfm-F	TTTGAGAGATATCTTCTTCTGTCTTGTAAC	
PBP1b-cfm-R	GAAAAGAAAGGGTTAATATCTTAGATGGGA	
PBP2-3×Flag-cfm-F	TGCGCCAGATCCTCGACCACATTATGCTGG	
PBP2-3×Flag-cfm-R	ATCCTGACCGCTGGCGCTCCAGATAACCAG	
PBP3-3×Flag-cfm-F	TGGGCGGCGTATTGCGTACCATGAACATCG	
PBP3-3×Flag-cfm-R	TACAAAGAGATCGCCCGCCGCAGCCACACG	
DacA(Δ)-3×Flag-cfm-F	GAAAGCCAGCTATGTGCTGA	
DacA(Δ)-3×Flag-cfm-R	TGATCAACCAGCTCAGGTAA	
pBAD-DacA-F	CTAGCAGGAGGAATTCATGAATACCATTTTTTCCGC	pBAD24 cloning

pBAD-DacA-R	GCAGGTCGACTCTAGATTTAACCAAACCAGTGATGG	
pBAD-DacC-F	CTAGCAGGAGGAATTCATGACGCAATACTCCTCTC	
pBAD-DacC-R	GCAGGTCGACTCTAGATTAAGAGAACCAGCTGCCG	
pBAD-DacD-F	CTAGCAGGAGGAATTCATGAAACGCCGTCTTATTATTGC	
pBAD-DacD-R	GCAGGTCGACTCTAGAAGAAAGGTCAGGCCTTATGGTGG	
pBAD-YfeW-F	CTAGCAGGAGGAATTCATGAAACGGACAATGCTCTA	
pBAD-YfeW-R	GCAGGTCGACTCTAGATTACTTCTGCTTTAACGCCG	
pBAD-insert-FRT-Km^r-F	GATGATGACGACAAATAGAGATTGCAGCATTACACGTC	pBAD-DacA(ΔC)-Flag-FRT-Km^r
pBAD-insert-FRT-Km^r-R	CAGCATCACCTCATCGCCATTAATTCACTG	
pBAD-Flag-FRT-Km^r-vF	GTAATGCTGCAATCTCTATTTGTCGTCATCATCTTTAT	
pBAD-frt-Km^r-vR	ATGGCGATGAGGTGATGCTGCTGCAGGCATGCAAGCTTGG	
pBAD-insert-DacA(ΔC)-FLAG-F	AGCAGGAGGAATTCCATATGAATACCATTTTTTCCGCTCG	
pBAD-insert-DacA(ΔC)-FLAG-R	AGGTCGACTCTAGACTCGAGGTTACCTTCCGGGATTTCTT	
pBAD-vector-DacA(ΔC)-FLAG-F	CATATGGAATTCCTCCTGCT	
pBAD-vector-DacA(ΔC)-FLAG-R	CTCGAGTCTAGAGTCGACCT	
pET24-DacA-F	AAGGAGATATACATATGGATGACCTGAATATCAAAAC	pET24- and pET28-
pET28-DacA-F	CGCGCGGCAGCCATATGGATGACCTGAATATCAAAAC	

pET-DacA-R	GCTCGAATTCGGATCCTTAACCAAACCAGTG ATGGAAC	DacA or DacA(ΔC) cloning
pET-DacA(ΔC)-R	GCTCGAATTCGGATCCTTAGTTACCTTCCGGG ATTTC	
pRE1-DacA-F	GGAAATACTTACATATGAATACCATTTTTTCC GCTCG	pRE1-DacA or DacA(ΔC) cloning
pRE1-DacA-R	GTCGACGATATCTAGATTAACCAAACCAGTG ATGGAAC	
pRE1-DacA(ΔC)-R	GTCGACGATATCTAGATTAGTTACCTTCCGG GATTTC	
DacA(S73G)-F	CCGCCGCGATCCTGCCGGCCTGACCAAAAT	Point mutant
DacA(S73G)-R	ATTTTGGTCAGGCCGGCAGGATCGCGGCGG	
DacA(K242R)-F	TGTCGACGGCATCAGAACCGGACACACTGA	
DacA(K242R)-R	TCAGTGTGTCCGGTTCTGATGCCGTCGACA	

References

Blattner FR, Plunkett G, 3rd, Bloch CA, Perna NT, Burland V, Riley M, Collado-Vides J, Glasner JD, Rode CK, Mayhew GF, Gregor J, Davis NW, Kirkpatrick HA, Goeden MA, Rose DJ, Mau B, Shao Y. 1997. The complete genome sequence of *Escherichia coli* K-12. *Science.* **277**:1453-1462. doi: https://doi.org/

Choi U, Lee CR. 2019. Distinct roles of outer membrane porins in antibiotic resistance and membrane integrity in *Escherichia coli*. *Frontiers in Microbiology.* **10**:953. doi: https://doi.org/10.3389/fmicb.2019.00953

Datsenko KA, Wanner BL. 2000. One-step inactivation of chromosomal genes in *Escherichia coli* K-12 using PCR products. *PNAS.* **97**:6640-6645. doi: https://doi.org/10.1073/pnas.120163297

Ji CJ, Yang YM, Kim JH, Ryu SH, Youn H, Lee JW. 2018. The roles of two O-donor ligands in the Fe^{2+}-binding and H_2O_2-sensing by the Fe^{2+}-dependent H_2O_2 sensor PerR. *Biochemical and Biophysical Research Communications.* **501**:458-464. doi: https://doi.org/10.1016/j.bbrc.2018.05.012

Park SH, Kim YJ, Lee HB, Seok YJ, Lee CR. 2020. Genetic evidence for distinct functions of peptidoglycan endopeptidases in *Escherichia coli*. *Frontiers in Microbiology.* **11**:565767. doi: https://doi.org/10.3389/fmicb.2020.565767

Reddy P, Peterkofsky A, McKenney K. 1989. Hyperexpression and purification of *Escherichia coli* adenylate cyclase using a vector designed for expression of lethal gene products. *Nucleic Acids Research.* **17**:10473-10488. doi: https://doi.org/10.1093/nar/17.24.10473
